# Determination of the Empirical Electrokinetic Equilibrium Condition of Microorganisms in Microfluidic Devices

**DOI:** 10.3390/bios10100148

**Published:** 2020-10-19

**Authors:** Adriana Coll De Peña, Nicole Hill, Blanca H. Lapizco-Encinas

**Affiliations:** 1 Microscale Bioseparations Laboratory and Biomedical Engineering Department, Rochester Institute of Technology, Rochester, NY 14623, USA; adrianacoll@brown.edu (A.C.D.P.); nsh3709@rit.edu (N.H.); 2 Thomas H. Gosnell School of Life Sciences, Rochester Institute of Technology, Rochester, NY 14623, USA

**Keywords:** electrokinetics, bacteriophages, electrophoresis, microfluidics, microorganisms

## Abstract

The increased concern regarding emerging pathogens and antibiotic resistance has drawn interest in the development of rapid and robust microfluidic techniques to analyze microorganisms. The novel parameter known as the electrokinetic equilibrium condition ($E_{EEC}$) was presented in recent studies, providing an approach to analyze microparticles in microchannels employing unique electrokinetic (EK) signatures. While the $E_{EEC}$ shows great promise, current estimation approaches can be time-consuming or heavily user-dependent for accurate values. The present contribution aims to analyze existing approaches for estimating this parameter and modify the process into an accurate yet simple technique for estimating the EK behavior of microorganisms in insulator-based microfluidic devices. The technique presented here yields the parameter called the *empirical* electrokinetic equilibrium condition ($eE_{EEC}$) which works well as a value for initial approximations of trapping conditions in insulator-based EK (iEK) microfluidic systems. A total of six types of microorganisms were analyzed in this study (three bacteria and three bacteriophages). The proposed approach estimated $eE_{EEC}$ values employing images of trapped microorganisms, yielding high reproducibility (SD 5.0–8.8%). Furthermore, stable trapping voltages (sTVs) were estimated from $eE_{EEC}$ values for distinct channel designs to test that this parameter is system-independent and good agreement was obtained when comparing estimated sTVs vs. experimental values (SD 0.3–19.6%). The encouraging results from this work were used to generate an EK library of data, available on our laboratory website. The data in this library can be used to design tailored iEK microfluidic devices for the analysis of microorganisms.

## 1. Introduction

Insulator-based electrokinetic (iEK) microfluidic techniques, especially dielectrophoresis (DEP), have been used for a large variety of bioanalytical applications [1]. Two primary modes of EK microfluidic techniques, electrode-based and insulator-based, have been used in numerous applications with DEP [2]. Electrode-based EK techniques are popular [3,4,5,6] and have demonstrated promising capabilities including the ability to analyze different strains of bacteria in a co-culture and track their behavior [7] and even categorize erythrocytes by their ABO-Rh blood types [8]. However, iEK microfluidic techniques are generally simpler to fabricate, inexpensive, disposable, and less affected to fouling than traditional electrode-based EK devices. Insulator-based EK microfluidic techniques have been used for the rapid identification, detection, and purification of both viruses [9,10,11] and cells [12,13,14,15], as these techniques can distinguish quickly between very similar organisms. For the analysis of cells, these techniques have been used to discriminate and separate between live and dead variations of the same cell type [16,17,18], bacteria of the same genus [12,13], differentiate serotypes of the same bacteria [15], and even distinguish between wild-type and antibiotic-resistant strains [19]. Individual subpopulations within a wider bacterial population have been separated by precise control of an alternating current (AC) signal and sharp insulating constrictions [20]. The ability to rapidly discriminate between bacterial cells down to the strain level by tracking their EK signature, which is difficult and time-consuming employing conventional techniques, could be crucial in selecting how to medicate a patient. Bacteriophage cocktails present a great potential for an alternative treatment to conventional antibiotics, but purification of bacteriophages is no easy feat. Purification of bacteriophages traditionally requires complex, labor intensive, and time-consuming processes with the potential to affect the stability and viability of the subjected bacteriophages [21], while EK microfluidic techniques have shown promise in distinguishing between bacteriophages while preserving their viability [9]. Expanding research into EK techniques might prove to be essential to advancing the development of analytical and purification techniques for bacteriophages. Additionally, it must be noted that while the focus of this study is the use of microscale EK techniques, microfluidic devices are currently being used across a wide range of fields. One such example, as presented by Baratchi et al. [22], is the in vitro study of cell mechanobiology, including the effects of shear stress on cells. These developments in iEK techniques could be combined with other cell characterization systems, such as the one presented by Baratchi et al. [22], which can result in a more comprehensive cell characterization process.

Numerous groups have utilized iEK microfluidic techniques for the study and manipulation of DNA [23,24,25], proteins [26,27,28], viruses [9,10,11], bacteria [13,15,19], yeast [29,30,31], mammalian cells [32,33,34,35], and even parasites [20]. All of these studies illustrate the growing interest in the development of iEK microfluidic systems for microorganism analysis. Moreover, the potential of iEK microfluidic techniques is further highlighted by the increased interest in fast and reliable diagnostics tools resulting from the COVID-19 pandemic as this technique has been successfully used to detect viruses [9,10,11]. Within these systems, EK has four main components: electroosmosis (EO), linear electrophoresis ($EP^{\left(1\right)}$), DEP, and non-linear electrophoresis of the second kind ($EP^{\left(3\right)}$). It must be noted, however, that while many of the reports cited above attribute a great extent of particle behavior at higher electric fields to DEP, publications from the physics field [36,37,38,39] and two recent reports [13,40] suggest that $EP^{\left(3\right)}$ may be the dominating mechanism of particle migration at high electric fields. Furthermore, one of these recent studies suggests that the contribution of the DEP to particle migration is low when compared to that of the $EP^{\left(3\right)}$ [13]. These findings may in part explain why mathematical models of iEK systems required the use of large correction factors associated with DEP in order to have agreement between model predictions and experimental results [41,42].

**Figure 1:**
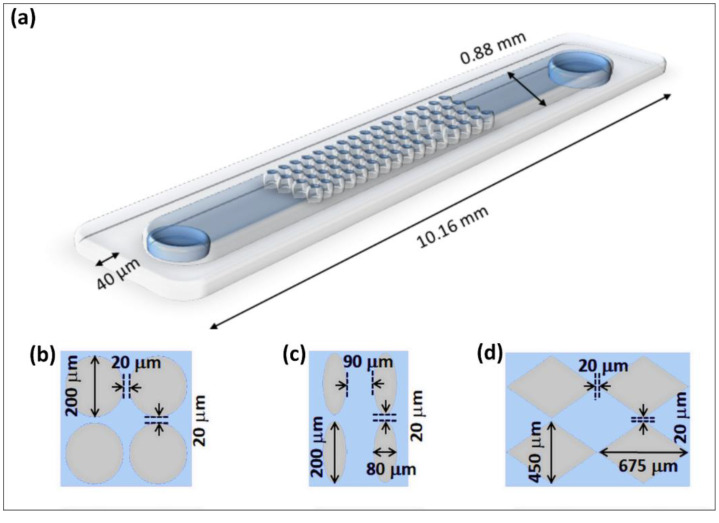
(**a**) Schematic representation of a microfluidic channel with post array. (**b**) Close-up of circle-shaped posts, (**c**) oval-shaped posts and (**d**) diamond-shaped posts.

By incorporating the effects of $EP^{\left(3\right)}$ in iEK systems, the parameter of electrokinetic equilibrium condition $\left(E_{EEC}\right)$ was proposed and estimated for polystyrene particles employing a post-less microchannel. In this device, particles exhibit three distinct behaviors as the electric field increases. First, particle velocity linearly increases with increasing electric fields. Second, particles reach a maximum velocity as a result of the competition between linear and nonlinear EK phenomena which shifts to a nonlinear relationship with electric fields. Third, particle velocity decreases as the non-linear EK phenomenon of $EP^{\left(3\right)}$ dominates particle migration. These three behaviors were demonstrated in the study by Cardenas-Benitez et al. [40], which introduced the parameter $E_{EEC.}$ This study was the first demonstration of particle trapping without insulating posts due to $EP^{\left(3\right)}$ effects [40]. The present contribution introduces a method for estimating a new parameter called the *empirical*-$E_{EEC}$, an offshoot of $E_{EEC}$ [40], determined in channels with insulating posts (Figure 1) to estimate trapping conditions in iEK microfluidic devices. The parameter $eE_{EEC}$ has the potential to be used as an EK signature for the rapid identification of microorganisms across different designs of iEK microfluidic devices. A total of six distinct microorganisms (three bacteria and three bacteriophage strains) were studied. The value of $eE_{EEC}$ for each microorganism was estimated in a straightforward manner by employing experimental images of microorganisms electrokinetically trapped between insulating posts, forming a stable band. We used the term stable trapping voltage (sTV) to identify the applied potential, which is higher than the traditional sufficient trapping voltage (TV), defined as the required voltage to obtain an *observable* band of trapped particles in iEK devices [9]. The use of channels with insulating post to determine $eE_{EEC}$ was presented in a recent preliminary study using bacterial and yeast cells [13]. The present contribution aims to review previous approaches, draw distinctions between $E_{EEC}$ and $eE_{EEC}$, and provide an accurate and simple method for estimating $eE_{EEC}$ and estimate electrokinetic trapping conditions. The results obtained demonstrated that once $eE_{EEC}$ has been determined for particular microorganism, this parameter can be used to estimate a starting range for the stable voltage (sTV) at which the microorganism will be trapped in any iEK microfluidic channel. Good agreement was found between estimated and experimentally determined sTV values. These encouraging results were used to generate an EK library of data, available on our laboratory website [43]. The $eE_{EEC}$ values presented here were obtained with a methodology that is simpler, faster, and less labor-intensive than the process for determining $E_{EEC}$ values [40]. The parameter $eE_{EEC}$ introduced here shows great potential for the rapid differentiation and identification of microorganisms in iEK microfluidic devices by employing $eE_{EEC}$ as a unique electrokinetic signature.

## 2. Theory

**Figure 2:**
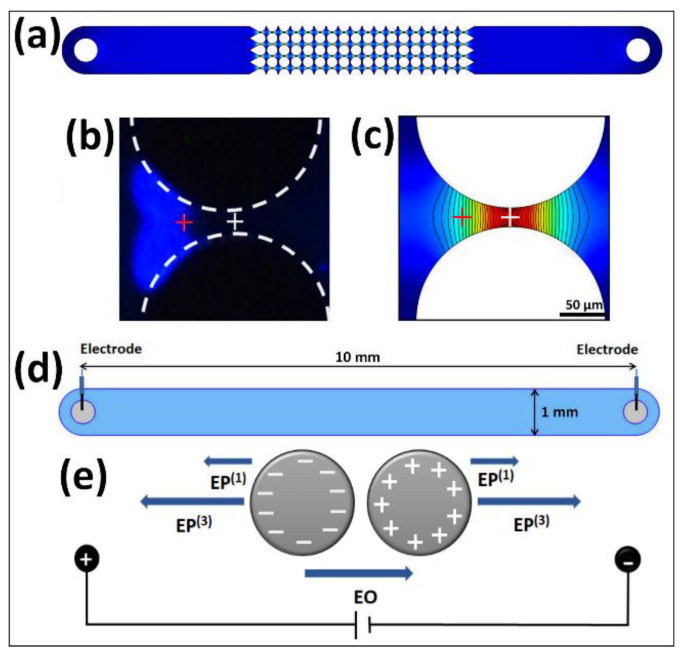
(**a**) COMSOL representation of the **E** distribution in a microchannel with circle posts. (**b**) Experimental image of *E. coli* cells trapping at 1000 V between two circle-shaped posts. (**c**) Distribution of **E** within the constriction region at 1000 V, where red represents the highest **E** magnitude. The black contour lines are isometric lines across which **E** is uniform, the red cross represents where the new method would estimate the *empirical* electrokinetic equilibrium condition ($eE_{EEC}$), and the white cross represents where the preliminary study [13] estimated the $eE_{EEC}$. (**d**) Schematic representation of post-less channel employed in the estimation of $E_{EEC}$ using particle velocimetry measurements [40]. (**e**) Forces acting on particles in post-less channels depending on their surface charges.

Electrokinetic phenomena are classified as linear and non-linear, determined by their dependence on the electric field [39]. Linear EK phenomena include EO, the flow of liquid relative to a stationary surface due to the electrical double layer (EDL) of the channel walls, and $EP^{\left(1\right)}$, the motion of electrically charged particles relative to a stationary fluid. The velocity expressions for EO and $EP^{\left(1\right)}$ are:

(1) $$v_{EO}=\mu_{EO}E=-\frac{\varepsilon_{m}\zeta_{W}}{\eta }E,$$

(2) $$v_{EP}^{\left(1\right)}=\mu_{EP}^{\left(1\right)}E=\frac{\varepsilon_{m}\zeta_{P}}{\eta }E,$$

where $\mu_{EO}$ and $\mu_{EP}^{\left(1\right)}$ represent the EO and $EP^{\left(1\right)}$ mobilities, respectively, $\zeta_{W}$ and $\zeta_{P}$ are the zeta potentials of the wall and particle, respectively, and $\varepsilon_{m}$ and η are the media permittivity and viscosity, respectively. Non-linear EK phenomena include DEP, particle motion due to polarization effects caused by a non-uniform electric field, and EP of the second kind $\left(EP^{\left(3\right)}\right)$, an electrophoretic response that appears at higher E [36,37,38,39]. The expressions for DEP, $EP^{\left(3\right)}$, and the total particle velocity ($v_{P}$) considering all four EK effects are:

(3) $$v_{DEP}=\mu_{DEP}\nabla E^{2}$$

(4) $$v_{EP}^{\left(3\right)}=\mu_{EP}^{\left(3\right)}\left(E\cdot E\right)E$$

(5) $$v_{P}=v_{EO}+v_{EP}^{\left(1\right)}+v_{DEP}+v_{EP}^{\left(3\right)}$$

where $\mu_{DEP}$ and $\mu_{EP}^{\left(3\right)}$ are the DEP and $EP^{\left(3\right)}$ mobilities, respectively. A recent study [13] performed in devices with insulating posts (Figure 1 and Figure 2a–c) suggested that the DEP contribution to total particle velocity, in comparison to the $EP^{\left(3\right)}$ contribution, is only 0.89–5.95%. Therefore, it was decided for simplification purposes to neglect DEP from the expression for total particle velocity, thus:

(6) $$v_{P}=v_{EO}+v_{EP}^{\left(1\right)}+v_{EP}^{\left(3\right)}$$

Equation (6) is also an accurate expression of particle velocity in a post-less microchannel (Figure 2d–e), as DEP is not present [40]. From this expression the parameter $E_{EEC}$, which is the **E** at which a particle stops migrating and is electrokinetically trapped ($v_{P}=0)$, can be derived [13,40]:

(7) $$0=v_{EO}+v_{EP}^{\left(1\right)}+v_{EP}^{\left(3\right)}=\mu_{EO}E+\mu_{EP}^{\left(1\right)}E+\mu_{EP}^{\left(3\right)}\left(E\cdot E\right)E$$

(8) $$E_{EEC}=\sqrt{-\frac{\left(\mu_{EP}^{\left(1\right)}+\mu_{EO}\right)}{\mu_{EP}^{\left(3\right)}}}$$

Equation (8), obtained by isolating E from the particle velocity equation, can be used to determine accurate $EP^{\left(3\right)}$ mobility data ($\mu_{EP}^{\left(3\right)}$) once $E_{EEC}$ has been identified from particle velocimetry measurements in post-less microchannels [40]. However, since significant experimentation and data analysis are required by numerous particle velocimetry measurements, the present study proposes the use of the *empirical*-$E_{EEC}$ ($eE_{EEC}$) which can be determined in much more straight forward manner. The $eE_{EEC}$ parameter is determined employing microchannels with insulating posts, where DEP forces are present and particle–particle interactions are more significant (due to higher particle concentrations) and influence particle trapping [44]. As DEP is still present in these channels, there is not a straightforward equation for $eE_{EEC}$ as there is for $E_{EEC}$. The $eE_{EEC}$ parameter offers a good estimation of the trapping voltages when testing microorganisms in an iEK system. Both $E_{EEC}$ and $eE_{EEC}$ are considered system-independent, but they are not media-independent, as the mobilities in Equation (8) depend on the suspending medium characteristics.

## 3. Materials and Methods

### 3.1. Microfluidic Devices

For this study, standard soft lithography techniques were used to cast polydimethylsiloxane (PDMS) microfluidic channels (Figure 1a) [13]. Upon curation with heat, the cast was removed from the mold, holes were punched to create channel liquid reservoirs and the cast was sealed with a PDMS-coated glass wafer using a plasma corona wand (Electro Technic Products, Chicago, IL, USA). This was done to ensure all the internal surfaces of the channel were made of the same material ensuring uniform EO flow. The devices were 10.16 mm long, 0.88 mm wide, and 40 µm tall. Microchannels with three distinct post shapes were made: circles, ovals and diamonds (Figure 1b–d).

### 3.2. Samples and Suspending Medium

For this study, three distinct bacteria species and three distinct bacteriophage species were used (Table 1). Size information for all microorganisms was measured in our lab or obtained from the literature [43]. These organisms were fluorescently labeled with one of the DNA intercalating Syto dyes, 11 or 45 (Invitrogen, Carlsbad, CA, USA). All organisms had a final concentration in the order of 10^6^ particles/mL or higher after labeling. The suspending media consisted of DI water purified using a RiOs™ 200 Water Purification System 120 V (MilliporeSigma, Burlington, MA, USA), and had a conductivity of 15.1 ± 6.1 μS/cm and a pH of 6.7 ± 0.5.

### 3.3. Equipment and Software

A Leica DMi8 inverted microscope (Wetzlar, Germany) paired with a Leica DFC7000 T camera and the software LASX provided by the manufacturer were used to record the behavior of the microorganisms. A high voltage supply (Model HVS6000D, LabSmith, Livermore, CA, USA) was used to apply direct current (DC) electric potentials. COMSOL Multiphysics^®^ 4.4 was used to simulate our system and estimate the $eE_{EEC}$ of each microorganism.

### 3.4. Experimental Procedure

A sample of 5–25 µL of the selected microorganism was introduced into the microfluidic channel with the desired post shape (Figure 1b–d). Platinum wires were placed at the inlet and outlet reservoirs and a DC electric potential was applied from left to right creating zones of higher field intensity within the insulating posts (Figure 2a). Voltages were gradually increased above TV to a voltage where stable bands were established, referred to as stable trapping voltage (sTV). Images of trapped microorganisms at sTV were captured and the sTV values were recorded. The location of the center of the “front” side of the band of trapped microorganisms was determined from the images, as depicted by the red cross in Figure 2b. This location at the “front” side of the band was selected because at this exact location is where particle velocity is zero, that is, all forces exerted on the microorganisms are in equilibrium. After the first microorganisms are trapped at this equilibrium location, additional microorganisms accumulate behind them, resulting in a band of trapped microorganisms. Therefore, the location of the “front” or inner side of the band is where the forces are in equilibrium, that is, the $eE_{EEC}$ value. These experiments were conducted in triplicate for each organism and each post shape. The sTV values were then input into COMSOL to simulate the distribution of E within the channel (Figure 2a) and the $eE_{EEC}$ was then estimated as the E at the location determined from the COMSOL image (red cross in Figure 2c), where $v_{P}=0$.

## 4. Results and Discussion

### 4.1. Discussion of Methods for Estimating $E_{EEC}$ in iEK Microfluidic Devices

Cardenas-Benitez et al. [40] was the first to estimate the novel parameter $E_{EEC}$ for four types of polystyrene particles using a post-less channel (Figure 2d) and particle tracking velocimetry (PTV) measurements. The forces acting on the particles in this system are illustrated in Figure 2e. Each particle type was run individually, and particle velocities were tracked at both low and high E regimes until particle motion switched directions, i.e., particles started to migrate backwards. When the particle switched directions, the **E** at which $v_{P}=0$ was recorded and used to estimate the $E_{EEC}$ for each particle. Therefore, numerous videos of each particle type had to be analyzed at different applied voltages to determine the $E_{EEC}$.

In a study by Coll De Peña et al. [13] that employed channels with insulating posts, the DEP contribution to the particle velocity was neglected (Equation (6)), as it was estimated to be between 0.89–5.95% in comparison to the $EP^{\left(3\right)}$ contribution. This study estimated $eE_{EEC}$, since microfluidic devices with posts were used, by combining experimental information with COMSOL estimations of the electric field distribution between posts (Figure 2a). Experiments in that preliminary study were focused on estimating the trapping voltage (TV), which is the applied voltage when the particle trapping becomes *observable*. Experiments with five strains of bacteria and one of yeast cells were performed in microfluidic devices containing circle and oval shaped posts (Figure 1c,d). Each organism was run separately in microchannels with each post shape, and the TV was visually determined, as the example shown in Figure 2b, by gradually increasing the applied voltage. Upon obtaining the TV experimentally, a COMSOL model was used to estimate E, which is equivalent to the $eE_{EEC}$ at $v_{P}=0$; E was estimated at the center of the constriction (where E is max), as represented with the white cross in Figure 2c.

The new approach presented here has two goals: (i) clearly distinguish between $E_{EEC}$ [40] and $eE_{EEC}$ [13] and (ii) accurately estimate the new parameter $eE_{EEC}$ by employing a new distinct location (red cross in Figure 2b,c) within the constriction region. The $E_{EEC}$ values presented in the seminal paper by Cardenas-Benitez et al. [40] are determined through significant experimental and data analysis work with polystyrene particles in devices without posts, ensuring that only three forces are present: $EP^{\left(1\right)}$, EO, and, at higher voltages, $EP^{\left(3\right)}$. Numerous experiments were needed to be performed at several different voltages, the resulting particle velocity data required extensive analysis, leading to a labor intensive and time-consuming process to properly determine the $E_{EEC}$ values. The seminal study by Cardenas-Benitez et al. [40] presented fundamentals of $E_{EEC}$ and $EP^{\left(3\right)}$ in iEK devices, as shown in Equation (8). The present contribution proposes a different approach, as it focuses instead on the application of experiments in iEK devices with insulating posts and higher particle concentrations. Higher particle concentrations lead to particle–particle interactions [44], and the presence of insulating posts leads to *DEP* effects, which can influence the $eE_{EEC}$ value. Bacterial cells and bacteriophages were used in the $eE_{EEC}$ estimations. While $E_{EEC}$ is the more accurate parameter, the $eE_{EEC}$ value is easier to obtain, requires much less experimental work, and can still provide valuable estimations that can be used as an EK signature for the rapid identification of particle and microorganisms. The $eE_{EEC}$ value is determined with a simpler, faster, and far less labor-intensive method that works well to provide an initial estimation of stable trapping voltages for different species. Another important advantage of employing $eE_{EEC}$ is that lower voltages are required, since the presence of insulating posts produces higher local electric fields; allowing reaching electrokinetic equilibrium at lower applied voltages than those required in PIV channels. A characterized species might be expected to have similar $E_{EEC}$ and $eE_{EEC}$ values, though the contribution of DEP and particle–particle interactions could potentially contribute to deviations in reported values. The goal of $eE_{EEC}$ values and addressing $eE_{EEC}$ and the technique to determine it is to give a good estimate for trapping conditions within an insulating electrokinetic microfluidic device. Addressing the second goal, between the preliminary study to determine $eE_{EEC}$ [13] and this study, the use of observable trapping voltage (TV) vs. stable trapping voltage (sTV) is the primary distinction. This is crucial as the TV can be affected by sample concentration given that a band may not be *observable* until highly concentrated, introducing subjectivity into the system. Thus, rather than estimating the $eE_{EEC}$ at the center of the constriction [13] (white cross, Figure 2b,c), the new location for estimating $eE_{EEC}$ was determined by experimental images at sTV values, i.e., when the band of trapped microorganism is clear and stable (red cross, Figure 2b,c). To emphasize this point, the $eE_{EEC}$ values from the prior preliminary study [13] for the bacteria were compared to the values obtained with the new approach presented here. All of the $eE_{EEC}$ values from the prior study were higher and had a higher variance (34–76%) than those found in this present study. It is clear from the findings of the present study that the new method proposed for estimating $eE_{EEC}$ is robust and reliable.

### 4.2. Estimations of the New $eE_{EEC}$ Parameter

**Figure 3:**
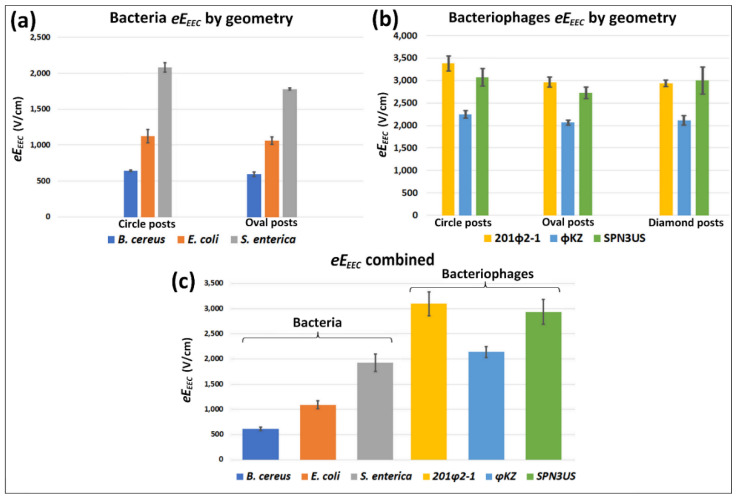
The $eE_{EEC}$ values are presented in each post shape per species for (**a**) the bacteria [13] and for (**b**) the bacteriophages [9]. (**c**) The $eE_{EEC}$ values obtained in each post shape were then averaged per species and are represented as a bar plot where the bacteria show lower overall values than the bacteriophages.

For every microorganism, experiments were conducted in triplicate in each post shape, and for each trial an image of the EK trapping of the microorganism at the sTV was used for estimating the $eE_{EEC}$ at the location illustrated in Figure 2b,c (red cross). The estimations of $eE_{EEC}$ are reported in Figure 3a,b for cells and bacteriophages, respectively. The average combined $eE_{EEC}$ for each distinct microorganism species across the distinct post shapes are shown in Figure 3c, where, as expected due to larger size, bacteria have lower $eE_{EEC}$ values than the bacteriophages [41]. The standard deviation across post shapes for all species ranged from 0.8 to 9.9%; in particular, it ranged from 0.8 to 8.3% for the bacteria (Figure 3a) and from 2.5 to 9.9% for the bacteriophages (Figure 3b).

The standard deviation of the estimation for the circle, oval, and diamond-shaped posts were 4.6%, 3.6%, and 5.8%, respectively. These low values suggest that this technique is not biased towards any particular post shape or feature. Thus, given that $eE_{EEC}$ is post shape-independent, the $eE_{EEC}$ values obtained for each microorganism in each post shape were then averaged together (Figure 3c and Table 1), yielding low standard deviations (5.2–8.8% for bacterial and 5.0–8.4% for bacteriophages). This low variability in $eE_{EEC}$ values illustrates the high precision and accuracy of this method. Considering that a portion of this variability could be introduced by neglecting the DEP contribution, and that biological samples have an innate population diversity, these variations are well within our tolerance. The EK library reflecting these results is publicly available on our laboratory website [43].

### 4.3. Application of the New $eE_{EEC}$ Parameter to Estimate Stable Trapping Voltages (sTVs)

**Figure 4:**
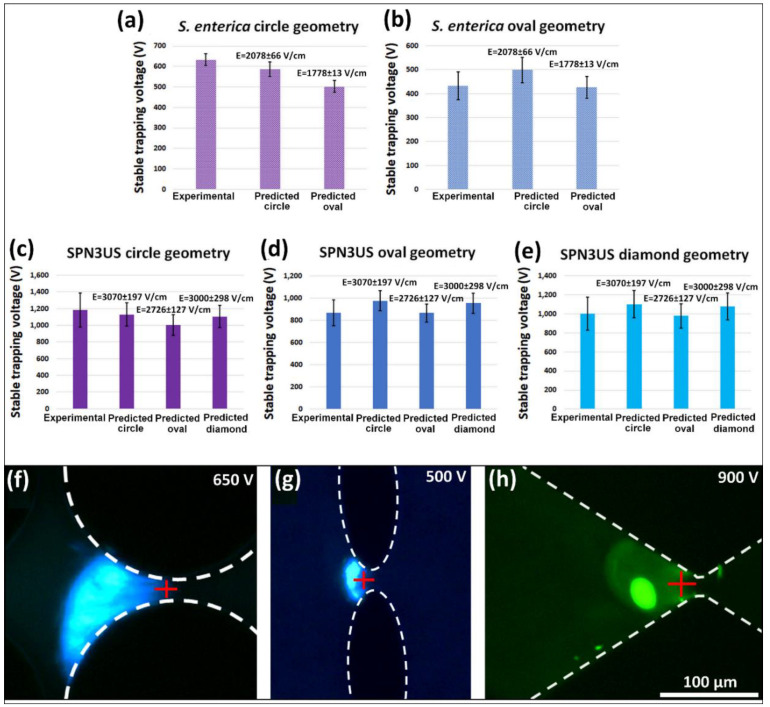
Comparison of experimental stable trapping voltages (sTVs) and estimated sTVs. Experimental and estimated sTVs for (**a**,**b**) *S. enterica* and for (**c**–**e**) SPN3US. All estimated sTV values were obtained using the $eE_{EEC}$ values reported in Figure 3 (reported above the relevant bars in the plots) and applying those $eE_{EEC}$ values to the geometry described in the title of the graphs. The experimental sTV values are the average applied voltages for the experimental images used to predict sTV. Estimations of sTV for *S. enterica* (**a**) in the circle posts, (**b**) in the oval posts. Estimations of sTV for SPN3US (**c**) in the circle posts, (**d**) in the oval posts and (**e**) in the diamond posts. (**f**–**h**) Images of trapped microorganisms illustrating the location used for estimations (red cross). Trapping of (**f**) *S. enterica* in circle posts at 650 V, (**g**) *S. enterica* in oval posts at 500 V, and (**h**) SPN3US in diamond posts at 900 V.

To demonstrate the applicability of the new $eE_{EEC}$ parameter for estimating sTV values in iEK microfluidic devices with different post shapes, the sTV values for one bacteria species (*S. enterica*) and one bacteriophage species (SPN3US) were estimated using COMSOL. The E values at the locations at which the sTV values were estimated for Figure 4a–e can be seen in Figure 3a,b. To vary these estimations, three distinct locations were determined for each post shape by employing the previously estimated location, plus and minus one standard deviation. In all the simulations, the third column of posts within the channel was used to perform a voltage sweep to determine the E values. As these E values were found at single positions, the E values were linearly dependent on the voltages. Linear interpolation was used to estimate the corresponding voltages to the $eE_{EEC}$ value for each distinct post shape. Then, the estimated voltages were compared with the experimentally obtained voltages. This illustrates the versatility of $eE_{EEC},$ since it can be applied to channels with other post shapes to estimate the applied voltage needed to achieve stable EK trapping of the microorganism at specific positions. The sTV values found at each position were averaged together; these values are reported in Figure 4a,b,f,g for bacteria *S. enterica* and Figure 4c–e,h for bacteriophage SPN3US. Experimental sTV values in Figure 4a–e are the averaged experimental sTV values for all images used. Estimated sTV values were obtained from the $eE_{EEC}$ values reported in Figure 3.

The standard deviation in the estimation of sTV ranged between 0.3% and 19.6% with an average of only 5.2% for *S. enterica* and 5.7% for SPN3US bacteriophage. Components of these deviations can be the accuracy for determining the band positions (represented by the red crosses in Figure 4f–h), variations in suspending media characteristics, microorganism population distribution, and the choice to neglect DEP effects. The applicability and good accuracy of this approach are supported by the little overlap that exists between the small error bars of the experimental and estimated sTV values in Figure 4b–e, suggesting that there is no significant statistical difference between experimental and estimated values. It is important to note that Figure 4a does show that the estimated sTV values based on the $eE_{EEC}$ value for the oval posts is outside of the error bar range for the experimental voltages. This is not the case for the results in Figure 4b–e, where estimated sTV values are within the error bars of the experimental sTV values. As expected from previous reports [41,45], the sTV values in the circle posts are the highest, followed very closely by the diamonds, when present, and then by the oval posts. This is consistent throughout several different experiments. Variations in the system might be the cause behind this discrepancy, but the lower values for oval posts versus the higher values for circle posts should also be noted. In general, this approach does demonstrate reasonable estimations for sTV value range. In addition, it can also be observed that the sTV values for SPN3US are significantly higher than those for *S. enterica* (Figure 4a–e), which is also expected based on their significant size difference. These anticipated trends further support the validity of this approach.

**Figure 5:**
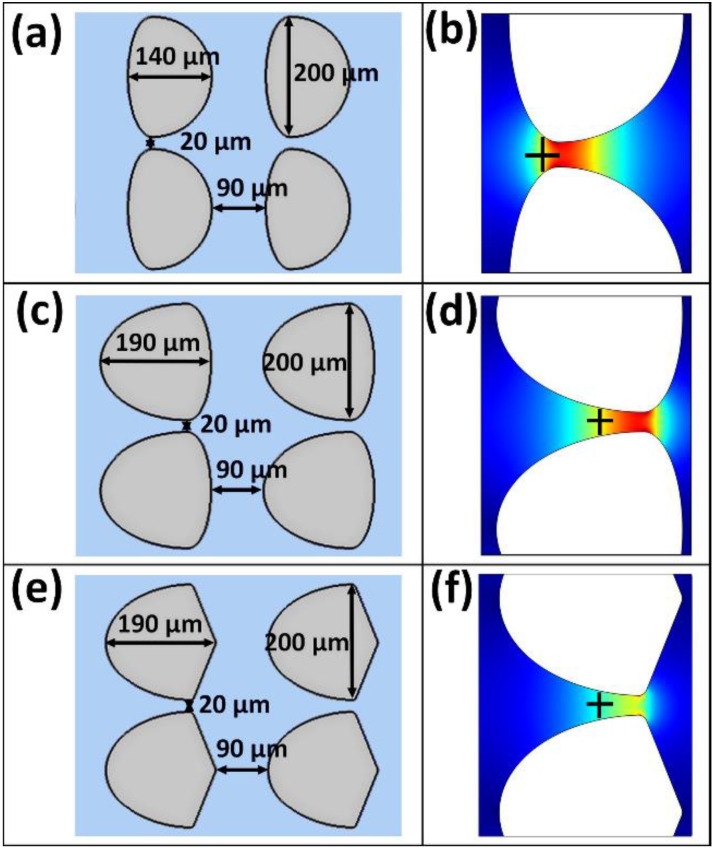
Schematic representation of the post dimensions and close-up of a constriction depicting the **E** distribution for three distinct post shapes: (**a**,**b**) oval–narrow–oval posts, (**c**,**d**) oval–wide–oval posts and (**e**,**f**) oval–wide–diamond posts. The color distribution represents the magnitude of **E** where red represents the highest **E** magnitude, and the black crosses represent the location at which the sTV values were estimated.

Taking this technique a step forward, its potential applicability is demonstrated by predicting potential sTV values in three extra post shapes from one of our recent studies [46]. Three distinct asymmetrical post shapes (Figure 5a,c,e) were used for predicting sTV values for *S. enterica* and SPN3US by employing their respective EK signature ($eE_{EEC}$). The specific positions considered for these estimations are marked with a black cross in Figure 5b,d,f, which were 25% of the length of the left-half of the constriction. This specific location was used because it is the average distance from the center in all images analyzed. For symmetric post shapes, the highest E value exists at the exact center of the constriction, where the vertical spacing is smallest. For asymmetric posts, the highest E value is very close to the center, where the vertical spacing is smallest and the posts divide. For consistency with our previous results, in all of these simulations, the third column of posts within the channel was used.

From Figure 5 it can be observed that the estimated sTV values were highest in the oval–wide–oval posts (Figure 5c,d), followed by the oval–wide–diamond posts (Figure 5e,f) and then by the oval–narrow–oval posts (Figure 5a,b) for both microorganisms as reported in Table 2. This trend agrees with theory as shown by a previous study involving these asymmetric posts [46]. As expected, the significantly larger microorganism, *S. enterica* bacterial cells, had lower predicted sTV values than the smaller SPN3US bacteriophages. While these values are modeling predictions only, they were estimated based on results from experimental data in other post shapes, and the trends observed in these predictions are encouraging. These results illustrate the ability to use $eE_{EEC}$ to predict sTV in alternate post shapes, which can be crucial in the design of more robust experiments where high resolution separations are required.

## 5. Conclusions

The present work illustrates a new methodology for estimating the empirical electrokinetic equilibrium condition ($eE_{EEC}$) in insulator-based devices containing posts. The EK behavior of three distinct species of bacteria and three species of bacteriophage viruses was analyzed. While there are prior approaches reporting estimations of $E_{EEC}$ [40] and $E_{EEC}$ values [13], both approaches have limitations. The particle velocimetry approach in post-less microchannels [40] is the most accurate and can be used to also estimate mobility data, but it is highly labor intensive. In an attempt to simplify this process, Coll De Peña et al. [13] presented the second approach in which experiments are conducted in microchannels with insulating posts and relies on determination of the trapping voltage (TV). This technique requires significantly less experimentation but has to contend with the impact of DEP and particle–particle interactions on the system, hence classifying it here as $eE_{EEC}$, rather than $E_{EEC}$. The goal of the present study is to improve the preliminary method for the determination of the $eE_{EEC}$ by utilizing images of bands of microorganisms electrokinetically trapped at the *stable* trapping voltage (sTV) for estimating the new $eE_{EEC}$ parameter. The present study properly introduces the new $eE_{EEC}$ parameter, which has a determination process that is simpler, faster, and less labor-intensive than that of the $E_{EEC}$ parameter determined in devices without insulating posts [40]. The approach presented here is more robust, as it has lower user-dependence than the previous report illustrating determination in devices with posts [13].

The results from this work illustrate good precision in the estimations of $eE_{EEC}$, with low standard deviations (<10%). The applicability $eE_{EEC}$ was further validated by performing estimations of sTV values for two microorganisms, bacteria *S. enterica* and bacteriophage SPN3US, which produced great agreement between the estimations and experimentally obtained sTV values (average species deviations < 6%). Moreover, the $eE_{EEC}$ values identified here were also used to predict the sTV values for three additional post shape designs to illustrate the versatility of $eE_{EEC}$ as a post shape-independent parameter. A summary of all the results obtained here, which are the unique EK signatures ($eE_{EEC}$) of these microorganisms can be found on our EK library available on our laboratory website [43]. The data in this library can be used to design iEK microfluidic devices for any applications that require the rapid identification of microorganisms.

## Figures and Tables

**Table 1:** List of all the microorganisms used in the study, along with their $eE_{EEC}$ and the standard deviation. All $eE_{EEC}$ values were estimated from experimental data.

Microorganism Type	Species	Size (µm)	$eE_{EEC}\;(V/\mathrm{cm})$	SD (%)
**Bacteria**	*Bacillus cereus* (ATCC^®^ 14579™)	**Length**: 4.94 ± 0.47 **Width**: 1.32 ± 0.13	618 ± 32	5.2
	*Escherichia coli* (ATCC^®^ 25922)	**Length**: 2.01 ± 0.42 **Width**: 0.97 ± 0.21	1092 ± 76	6.9
	*Salmonella enterica* (TT9079)	**Length**: 2.00 ± 0.31 **Width**: 0.97 ± 0.11	1928 ± 170	8.8
**Bacteriophages**	201Φ2-1	**Head dia.**: 0.200 **Tail**: 0.020 × 0.211	3094 ± 238	7.7
	ΦKZ	**Head dia.**: 0.145 **Tail**: 0.022 × 0.200	2140 ± 107	5.0
	SPN3US	**Head dia.**: 0.060 **Tail**: 0.018 × 0.035	2932 ± 246	8.4

**Table 2:** Predicted sTV values for *S. enterica* and SPN3US in each insulator-based electrokinetic (iEK) microfluidic channel.

Species	iEK Microfluidic Channel	Predicted sTV (V)
***S. enterica***	Oval–narrow–oval (Figure 5a,b)	519
	Oval–wide–oval (Figure 5c,d)	542
	Oval–wide–diamond (Figure 5e,f)	535
**SPN3US**	Oval–narrow–oval (Figure 5a,b)	780
	Oval–wide–oval (Figure 5c,d)	815
	Oval–wide–diamond (Figure 5e,f)	805
